# Radiomics for the Preoperative Evaluation of Microvascular Invasion in Hepatocellular Carcinoma: A Meta-Analysis

**DOI:** 10.3389/fonc.2022.831996

**Published:** 2022-04-07

**Authors:** Liujun Li, Chaoqun Wu, Yongquan Huang, Jiaxin Chen, Dalin Ye, Zhongzhen Su

**Affiliations:** ^1^ Department of Ultrasound, The Fifth Affiliated Hospital of Sun Yat-Sen University, Zhuhai, China; ^2^ Guangdong Provincial Key Laboratory of Biomedical Imaging and Guangdong Provincial Engineering Research Center of Molecular Imaging, The Fifth Affiliated Hospital of Sun Yat-sen University, Zhuhai, China

**Keywords:** radiomics, microvascular invasion, hepatocellular carcinoma, diagnosis, meta-analysis

## Abstract

**Background:**

Microvascular invasion (MVI) is an independent risk factor for postoperative recurrence of hepatocellular carcinoma (HCC). To perform a meta-analysis to investigate the diagnostic performance of radiomics for the preoperative evaluation of MVI in HCC and the effect of potential factors.

**Materials and Methods:**

A systematic literature search was performed in PubMed, Embase, and the Cochrane Library for studies focusing on the preoperative evaluation of MVI in HCC with radiomics methods. Data extraction and quality assessment of the retrieved studies were performed. Statistical analysis included data pooling, heterogeneity testing and forest plot construction. Meta-regression and subgroup analyses were performed to reveal the effect of potential explanatory factors [design, combination of clinical factors, imaging modality, number of participants, and Quality Assessment of Diagnostic Accuracy Studies 2 (QUADAS-2) applicability risk] on the diagnostic performance.

**Results:**

Twenty-two studies with 4,129 patients focusing on radiomics for the preoperative prediction of MVI in HCC were included. The pooled sensitivity, specificity and area under the receiver operating characteristic curve (AUC) were 84% (95% CI: 81, 87), 83% (95% CI: 78, 87) and 0.90 (95% CI: 0.87, 0.92). Substantial heterogeneity was observed among the studies (*I²*=94%, 95% CI: 88, 99). Meta-regression showed that all investigative covariates contributed to the heterogeneity in the sensitivity analysis (*P* < 0.05). Combined clinical factors, MRI, CT and number of participants contributed to the heterogeneity in the specificity analysis (*P* < 0.05). Subgroup analysis showed that the pooled sensitivity, specificity and AUC estimates were similar among studies with CT or MRI.

**Conclusion:**

Radiomics is a promising noninvasive method that has high preoperative diagnostic performance for MVI status. Radiomics based on CT and MRI had a comparable predictive performance for MVI in HCC. Prospective, large-scale and multicenter studies with radiomics methods will improve the diagnostic power for MVI in the future.

**Systematic Review Registration:**

https://www.crd.york.ac.uk/prospero/display_record.php?RecordID=259363, identifier CRD42021259363.

## Introduction

Hepatocellular carcinoma (HCC) is the fourth leading cause of cancer-related deaths and the second most lethal tumor (1), with 905,677 new cases and 830,180 new deaths in 2020 worldwide (2). New HCC cases and deaths in China account for approximately 50% of the cases in the world (3). Surgical and local ablative therapies are recognized as the radical treatment for HCC (4). However, the postoperative 5-year recurrence rate is still as high as 70% (5). Studies have shown the relationship between the high recurrence rate and microvascular invasion (MVI), which is recognized as an independent risk factor for postoperative recurrence of HCC (6–8). Postoperative pathology is the gold standard for MVI, but it is a lagging indicator. Therefore, the preoperative evaluation of MVI status will contribute to inform decision-making about the extent of surgical resection or ablation treatment for patients with HCC.

Biopsy is the preoperative reference standard for the diagnosis of MVI. Nevertheless, it is an invasive operation that may cause correlative complications and tumor seeding (9). In addition, there are some false negative results due to specimen limitation and tumor heterogeneity. Therefore, a noninvasive evaluation system is needed for preoperatively identifying MVI. Ultrasound (US), computed tomography (CT), and magnetic resonance imaging (MRI) have been used to assess the MVI status of HCC based on morphological features, such as size, number, shape, boundary, edge, capsule and enhancement characteristics (10–12). Unfortunately, the results have been inconsistent. The reasons are that the spatial resolution is too low to detect microvessels, and the reviews of medical images rely on subjective experience. Thus, there is an unmet clinical need to objectively, standardly and quantitatively evaluate the MVI status of HCC.

In 2012, Dutch scholar Lambin (13) proposed the concept of radiomics. It could extract massive quantitative imaging features from medical images by the statistics methods or machine learning algorithms. Radiomics has been used to construct predictive models for MVI by extracting quantitative features from US, CT, MRI, or positron emission tomography (PET). However, these studies differed in the diagnostic performance of the preoperative evaluation of MVI due to the differences in imaging modalities, research methods, sample size and so on. The reported diagnostic power ranged from 68% to 98% in the above studies (14–35). For these reasons, the diagnostic performance of radiomics for the preoperative identification of MVI in clinical practice remains uncertain. Therefore, we collected relevant studies and performed this meta-analysis to investigate the diagnostic performance of radiomics for the preoperative evaluation of MVI in HCC and the effect of potential factors.

## Materials and Methods

### Literature Search and Study Selection

The present study followed the Preferred Reporting Items for Systematic reviews and Meta-Analysis of Diagnostic Test Accuracy (PRISMA-DTA) (36), and it was registered in the International Prospective Register of Systematic Reviews (number CRD42021259363). All papers were screened independently by two authors (LL and CW, radiologists with 8 and 2 years of experience, respectively). Any disagreements were resolved by discussion, and if the disagreement could not be resolved, a consensus was reached through arbitration by a third reviewer (YH, a radiologist with 11 years of experience).

We selected published relevant studies by systematically searching the PubMed, Embase and Cochrane Library databases without language, nation, or time restrictions. The deadline for searching the databases was June 06, 2021. We used subject words and free words such as “hepatocellular carcinoma”, “microvascular invasion”, and “radiomics” and their variations. A detailed search strategy is described in the **Supplementary Materials**. After the elimination of duplicate papers, the titles and abstracts of all remaining articles were reviewed. When it was ambiguous whether an article should be included, the full-text content had to be accessible online or in print and reviewed. Furthermore, we scrutinized the reference lists of each identified primary study and previous systematic reviews to identify additional related articles.

The inclusion criteria were as follows: diagnosis of HCC by pathology after hepatectomy or liver transplantation; presence or absence of MVI by pathologic diagnosis; US, CT, MRI or PET-CT performed one month before surgery; and imaging analysis based on a radiomics algorithm. The exclusion criteria were as follows: antitumor therapy was performed preoperatively; the studies did not have enough information to construct a two-by-two contingency table; the type of study included animal experiments, nondiagnostic tests, case reports, reviews, expert opinions and conference abstracts.

### Data Extraction and Quality Assessment

Data extraction and quality assessment of the retrieved studies were completed by two authors independently (LL and JC, radiologists with 8 and 5 years of experience, respectively). Any discrepancies were resolved by consensus with a senior author (ZS, a radiologist with 22 years of experience). We extracted data on patient characteristics, imaging modalities, and study characteristics from each selected study. Patient characteristics included the total number of participants, the number of participants with MVI present and MVI absent, sensitivity and specificity. We tabulated the number of true positives (TPs), false positives (FPs), false negatives (FNs) and true negatives (TNs) by the number of MVI-present and MVI-absent cases, sensitivity and specificity reported in each included study. If there were two or more predictive models based on the same cohort of patients in one study, the best model reported in the study was included in our meta-analysis.

We evaluated the methodological quality of the included studies by using the standard Quality Assessment of Diagnostic Accuracy Studies 2 (QUADAS-2) tool (Bristol University, Bristol, UK) (37). We followed the guidelines for scoring each item of the checklist to assess the risk of bias and concerns regarding applicability by the software Review Manager 5.4 (Cochrane Library Software, Oxford, UK). The four domains assessed are as follows: patient selection, index test, reference standard, and flow and timing. Each individual question was categorized as “yes”, “no” or “unclear” for the risk of bias and “high risk”, “low risk” or “unclear risk” for applicability concerns.

### Statistical Analysis

We used the MIDAS module for STATA version 16 (Stata Corp LP, College Station, Texas, USA) to analyze the raw data. We calculated the pooled sensitivity, specificity, positive likelihood ratio (PLR), negative likelihood ratio (NLR), and diagnostic odds ratio (DOR) and their corresponding 95% confidence intervals (CI) using a bivariate regression model. Moreover, we plotted the results on a summary receiver operating characteristic (SROC) curve, and the area under the receiver operating characteristic curve (AUC) exhibited the diagnostic power of the included studies (38). AUCs of 0.5–0.7, 0.7–0.9 and > 0.9 show low, moderate and high diagnostic value, respectively. In addition, we plotted the Fagan nomogram by the MIDAS module and used the pretest probability (the ratio of MVI-positive cases to all cases in the included studies), PLR and NLR to calculate the posttest probability.

We drew forest plots to show the variation among studies and to detect heterogeneity for the pooled sensitivity and specificity. Heterogeneity due to the threshold effect was tested with the STATA MIDAS module. Heterogeneity caused by nonthreshold effects was measured using Cochrane’s Q-test and inconsistency index *I^2^*, and the difference was considered significant when *P* < 0.05, with *I^2^* ≥ 50% regarded as being indicative of moderate-to-high heterogeneity among studies (39). Meta-regression and subgroup analysis were performed to investigate the potential sources of heterogeneity. We performed univariable meta-regression analysis of several relevant covariates: design (retrospective or prospective), combined clinical factors (yes or no), imaging modality (MRI, CT or US), number of participants (≥100 or <100), and QUADAS-2 applicability risk (absence or presence of high risk). In addition, the possible presence of publication bias was further assessed by using a Deeks’ regression test of asymmetry (40). Slope coefficients with a *P* value < 0.10 indicate significant publication bias.

## Results

### Literature Selection

**Figure 1:**
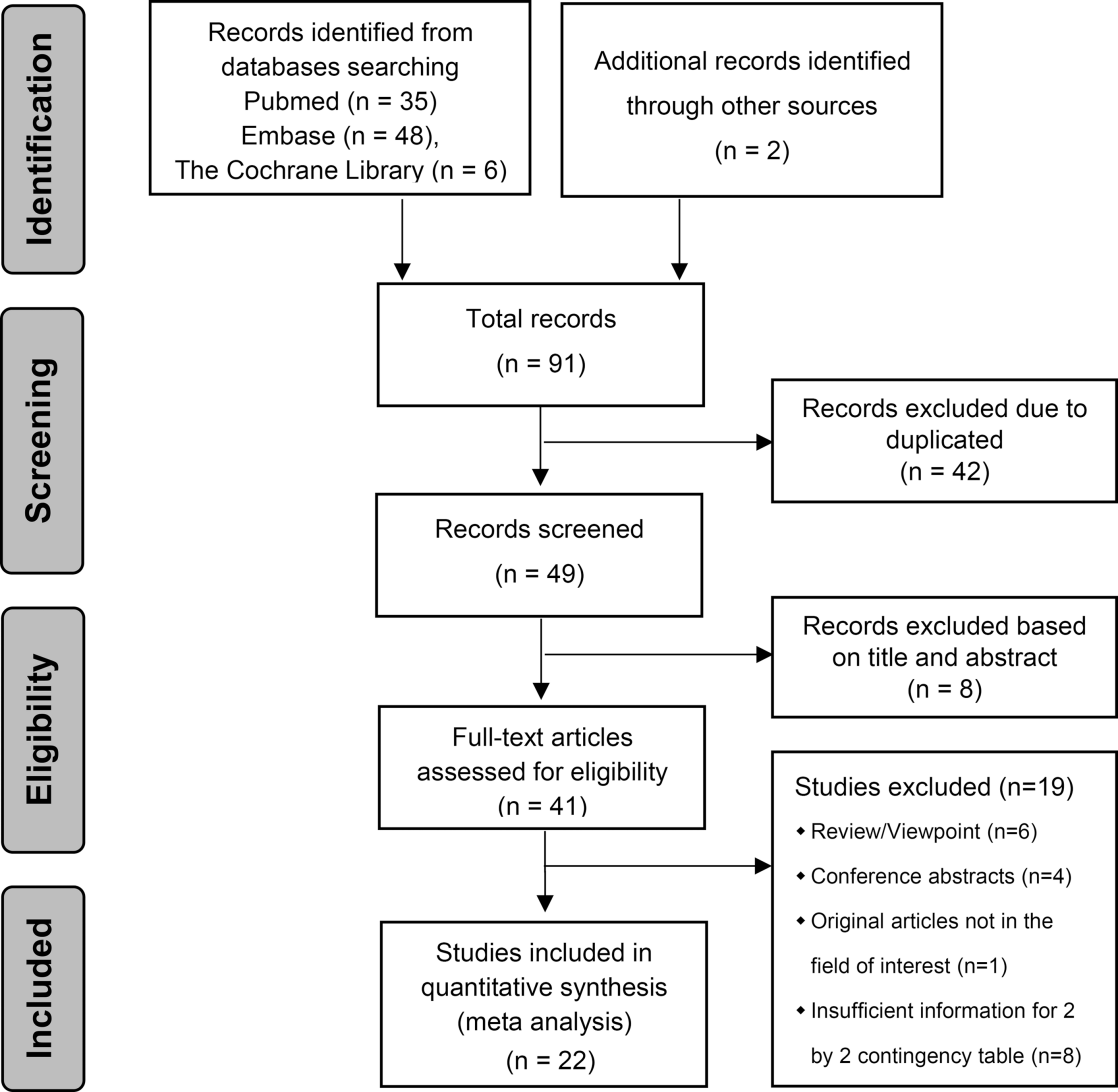
PRISMA flowchart of the study selection procedure.

The literature search and study selection are shown in **Figure 1** (36). All included studies were published between 2017 and 2021, and nine radiomics studies based on CT, nine radiomics studies based on MRI, three radiomics studies based on US and one radiomics study based on PET-CT were eligible for inclusion in this meta-analysis. A total of 4,129 HCC patients were included. Among them, 1,668 (40.4%) patients were pathologically diagnosed as MVI-positive and 2,461 (59.6%) as MVI-negative.

### Extracted and Quality Assessment

**Figure 2:**
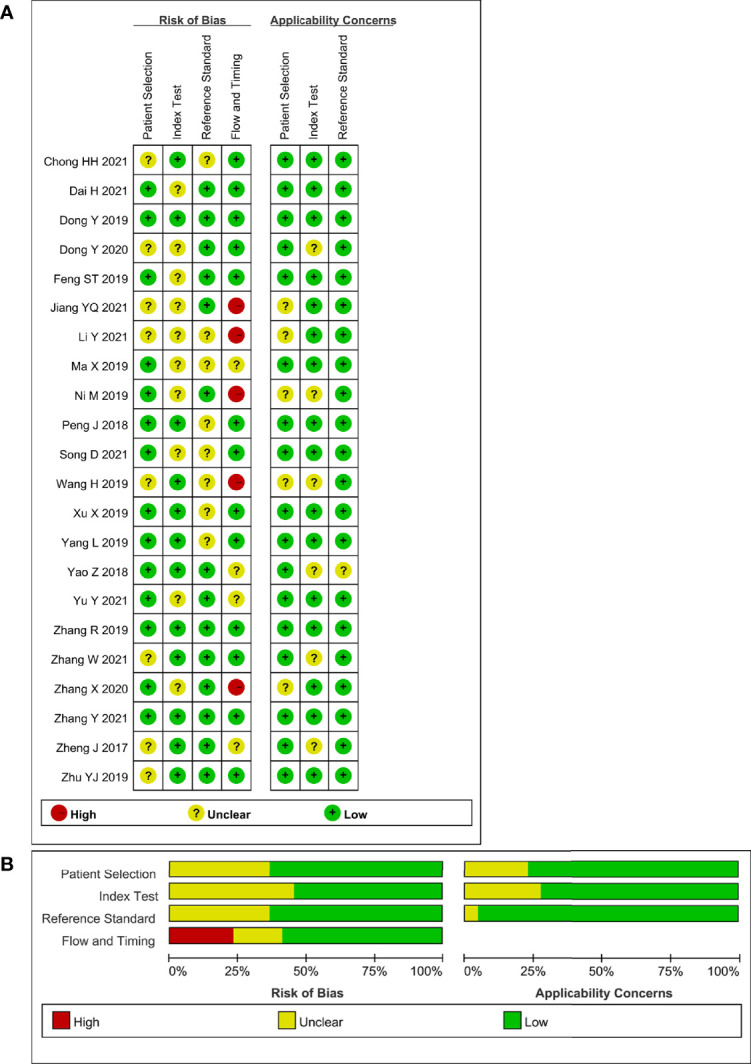
Stacked bar charts of the QUADAS-2 scale of methodological quality assessment. Risk of bias and applicability concerns of each included study. **(A)** Individual studies, **(B)** summary. For each quality domain, the proportions of included studies that suggest low, high, or unclear risk of bias and applicability concerns are displayed in green, red and yellow, respectively.

**Figure 3:**
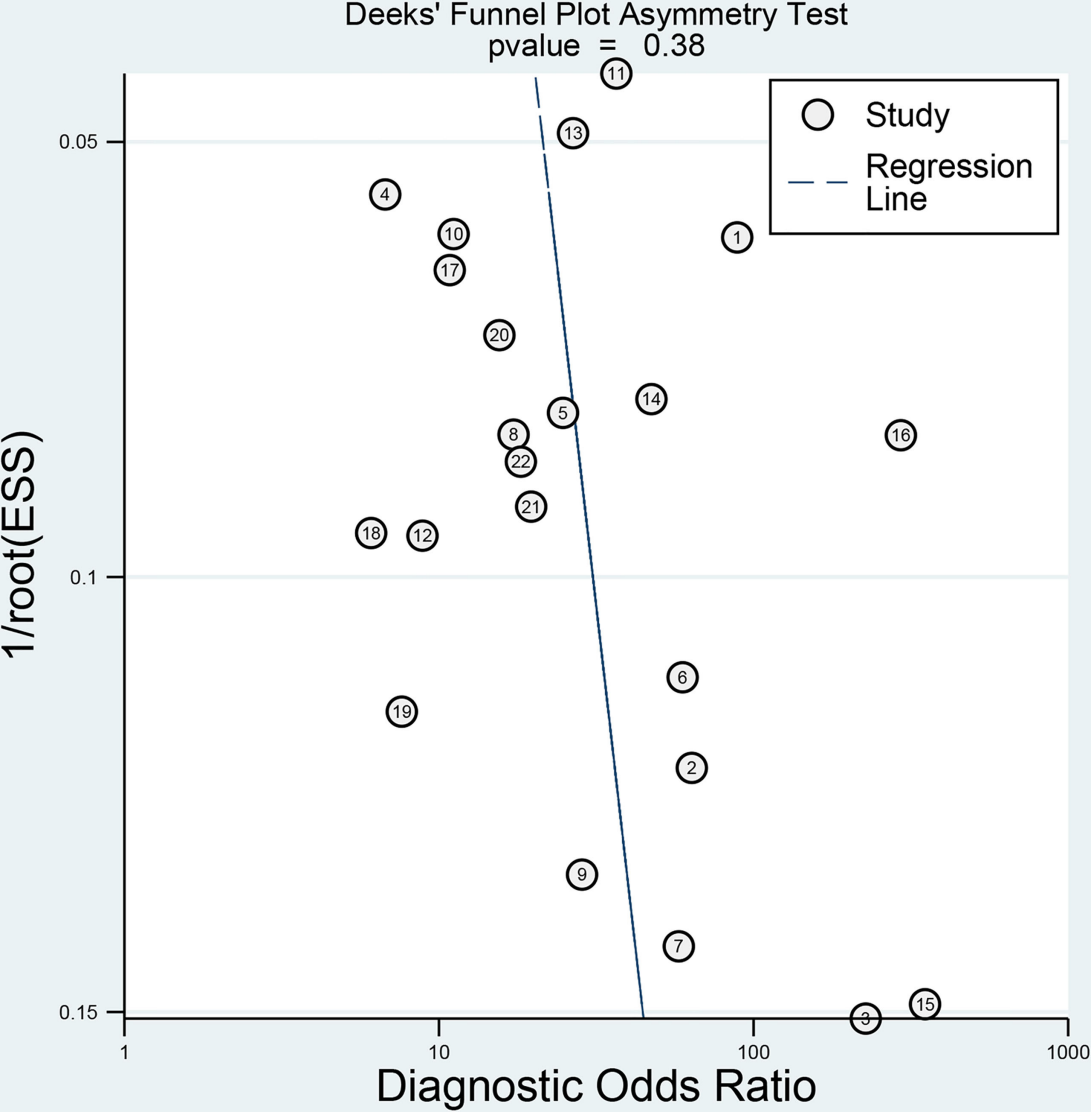
Deeks’ funnel plot shows no asymmetry and the presence of publication bias. Numbers in circles refer to the study ID. ESS, effective sample size.

The relevant characteristics and details of the 22 included studies are shown in **Table 1**. **Figure 2** displays the distribution based on the QUADAS-2 scale of the methodological quality assessment of the included studies. The majority of studies were judged to have a low risk of bias and minimal concerns regarding applicability. None of the studies were excluded from the analysis according to the quality assessment. The slope coefficients of Deeks’ funnel plot asymmetry test for the presence of publication bias (*P*=.38) are presented in **Figure 3**, which suggested no publication bias.

**Table 1:** Basic characteristics and details of the 22 included studies.

Study ID	First Author	Year	N	MVI-Present	MVI-Absent	TP	FP	FN	TN	Imaging Modality	Design	Combine Clinical Factors (Yes/No)	Cohort Detail
1	Chong et al. (14)	2021	356	90	266	80	22	10	244	MRI	retrospective	Yes	Training and Validation cohort
2	Dai et al. (15)	2021	69	29	40	27	7	2	33	MRI	retrospective	No	/
3	Dong et al. (16)	2019	42	21	21	18	0	3	21	US	prospective	No	/
4	Dong et al. (17)	2020	322	144	178	121	78	23	100	US	retrospective	No	/
5	Feng et al. (18)	2019	160	62	98	47	11	15	87	MRI	retrospective	No	Training and Validation cohort
6	Jiang et al. (19)	2021	81	44	37	34	2	10	35	CT	retrospective	Yes	Validation cohort
7	Li et al. (20)	2021	50	22	28	15	1	7	27	PET-CT	retrospective	No	Training cohort
8	Ma et al. (21)	2019	157	55	102	48	29	7	73	CT	retrospective	Yes	Training and Validation cohort
9	Ni et al. (22)	2019	58	23	35	19	5	4	30	CT	retrospective	No	Validation cohort
10	Peng et al. (23)	2018	304	201	103	157	25	44	78	CT	retrospective	Yes	Training and Validation cohort
11	Song et al. (24)	2021	601	225	376	191	50	34	326	MRI	retrospective	Yes	Training and Validation cohort
12	Wang et al. (25)	2019	125	41	84	29	18	12	66	MRI	retrospective	No	Test cohort
13	Xu et al. (26)	2019	495	149	346	132	78	17	268	CT	retrospective	Yes	Training/Validation and Test cohort
14	Yang et al. (27)	2019	208	53	155	47	22	6	133	MRI	retrospective	Yes	Training and Validation cohort
15	Yao et al. (28)	2018	43	21	22	19	0	2	22	US	prospective	No	/
16	Yu et al. (29)	2021	148	88	60	84	4	4	56	CT	retrospective	No	Training and Validation cohort
17	Zhang et al. (30)	2019	267	90	177	74	53	16	124	MRI	retrospective	Yes	Training and Validation cohort
18	Zhang et al. (31)	2021	111	57	54	41	16	16	38	CT	retrospective	No	Training/Validation and Test cohort
19	Zhang et al. (32)	2020	75	37	38	26	9	11	29	CT	retrospective	Yes	Validation cohort
20	Zhang et al. (33)	2021	195	110	85	91	20	19	65	MRI	retrospective	Yes	Training and Validation cohort
21	Zheng et al. (34)	2017	120	53	67	48	22	5	45	CT	retrospective	Yes	Tumor size: ≤ 5 cm and >5cm
22	Zhu et al. (35)	2019	142	53	89	43	17	10	72	MRI	retrospective	Yes	Training and Validation cohort

N, nubmer of patients; MVI, microvascular invasion; TP, true positive; FP, false positive; TN, true negative; FN, false negative; US, ultrasound; CT, computed tomography; MRI, magnetic resonance imaging; PET, positron emission tomography.

### Data Analysis

**Figure 4:**
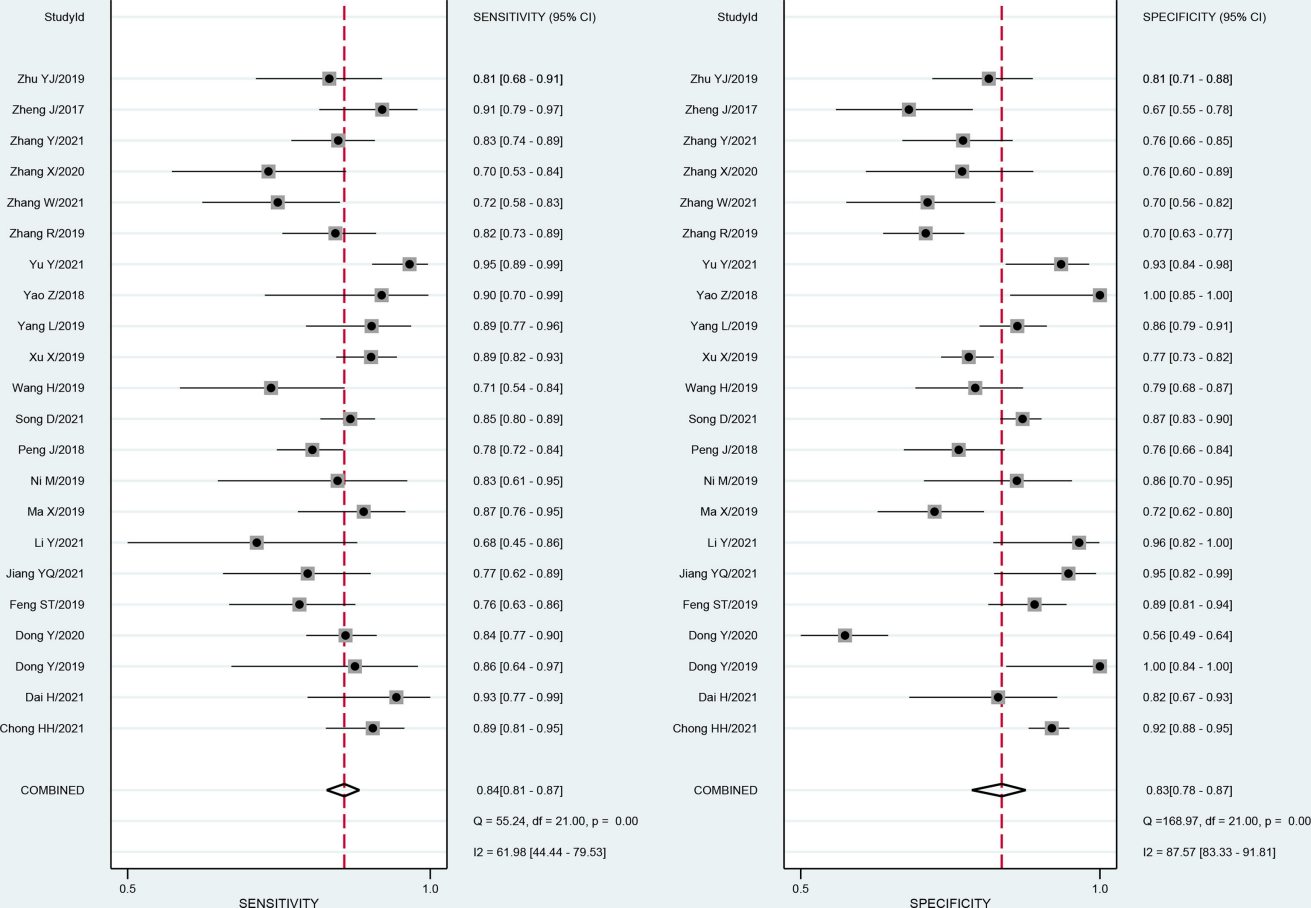
Forest plots show the performance estimates (sensitivity and specificity) of each study based on radiomics for the preoperative prediction of MVI in HCC. Vertical lines in the forest plots show the pooled estimates of sensitivity and specificity. I^2^ > 50% indicates substantial heterogeneity in the diagnostic parameters across studies.

**Figure 5:**
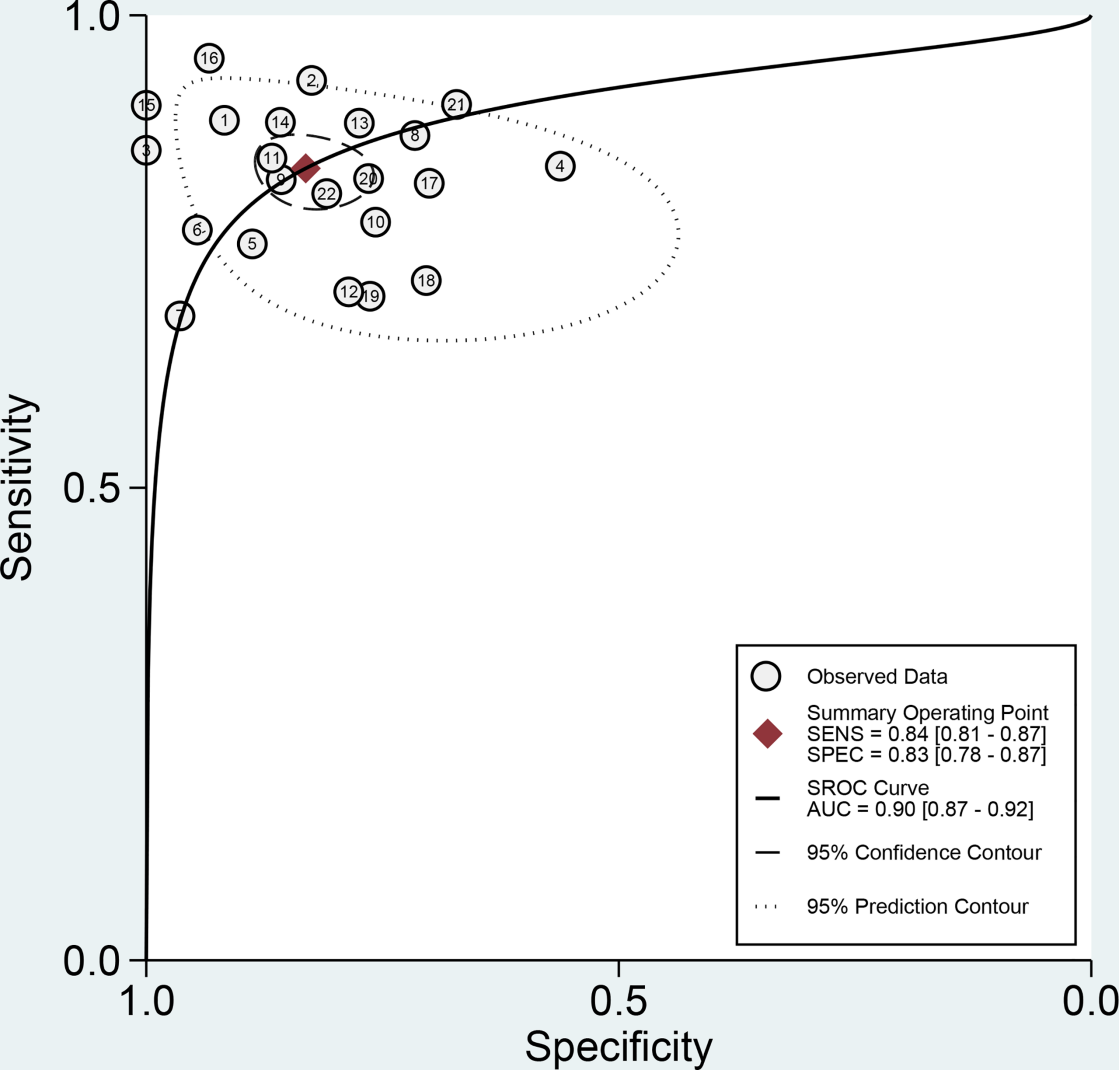
Summary receiver operating characteristic (SROC) plots of radiomics for the preoperative identification of microvascular invasion in hepatocellular carcinoma. Each circle indicates one included study. Values in brackets are 95% CIs. AUC, area under the receiver operating characteristic curve.

**Figure 6:**
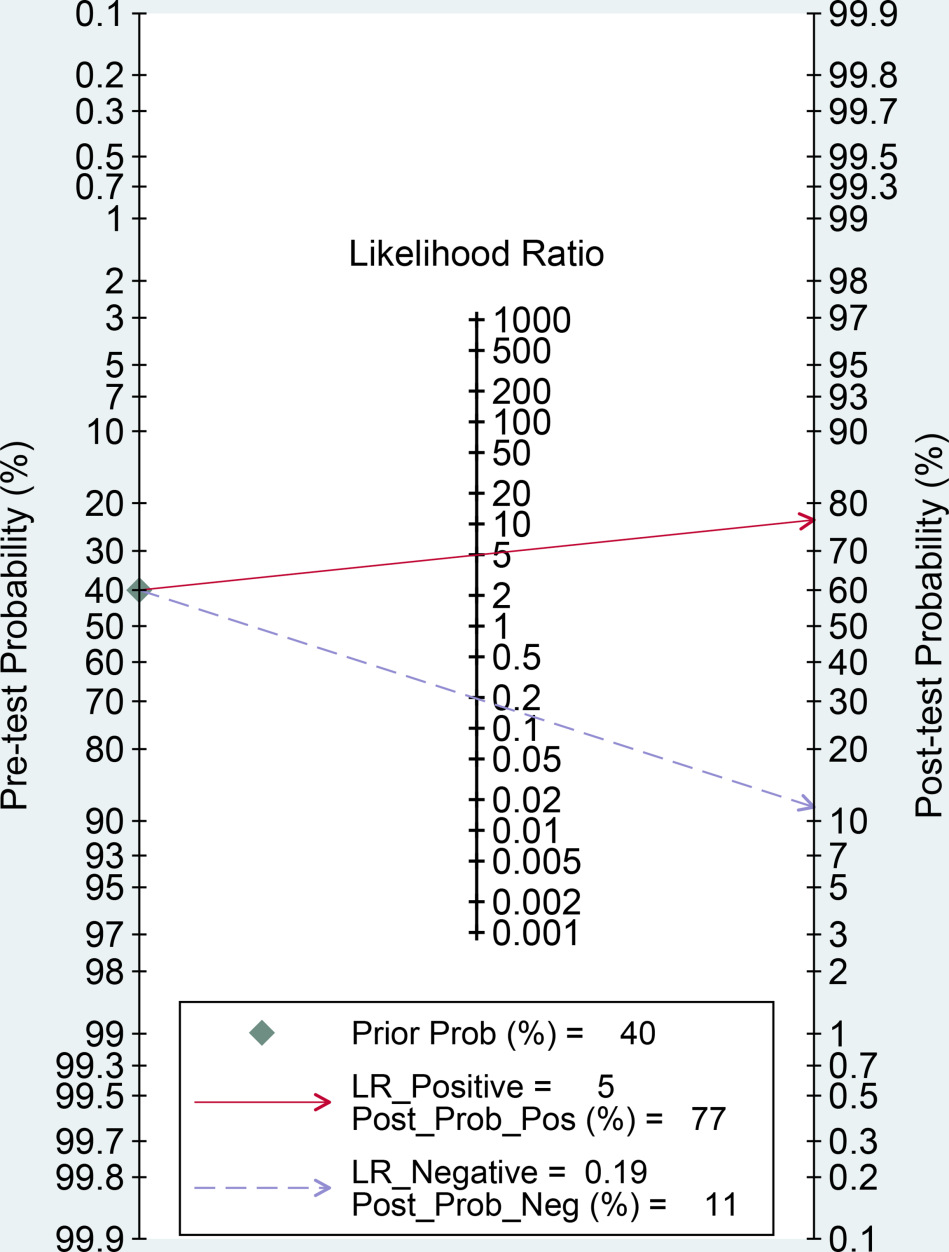
Fagan nomogram of radiomics for the preoperative identification of microvascular invasion in hepatocellular carcinoma. LR, likelihood ratio; Prob, probability; Pos, positive; Neg, negative.

The forest plots and comprehensive results of all studies included in this diagnostic meta-analysis are shown in **Figure 4** and **Table 2**. The primary analysis showed that the pooled sensitivity, specificity, PLR, NLR and DOR for the preoperative prediction of MVI in HCC were 84% (95% CI: 81%, 87%), 83% (95% CI: 78, 87), 5.0 (95% CI: 3.7, 6.6), 0.19 (95% CI: 0.16, 0.24) and 25.5 (95% CI: 16.7, 39.0), respectively. The AUC was 0.90 (95% CI: 0.87, 0.92), which suggested high diagnostic value (**Figure 5**). In addition, the pretest probability of MVI positive was 0.40 in our study, and both the likelihood ratio and posttest probability were high. A PLR of 5 implies an increase in the posttest probability for a positive test result to 77%. Likewise, an NLR of 0.19 reduced the posttest probability to 11% for a negative test result (**Figure 6**).

**Table 2:** Sensitivity analysis based on radiomics for the preoperative prediction of microvascular invasion in hepatocellular carcinoma.

Author	ACC (%)	SEN (%)	SPE (%)	PLR	NLR	DOR
Chong HH	91	89 (81, 95)	92 (88, 95)	10.75 (7.16, 16.14)	0.12 (0.07, 0.22)	88.7 (40.3, 195.3)
Dai H	87	93 (77, 99)	82 (67, 93)	5.32 (2.69, 10.50)	0.08 (0.02, 0.32)	63.6 (12.2, 332.0)
Dong Y^*^	93	86 (64, 97)	100 (84, 100)	37.00 (2.37, 576.55)	0.16 (0.06, 0.43)	227.3 (11.0, 1000.0)
Dong Y^†^	69	84 (77, 90)	56 (49, 64)	1.92 (1.60, 2.30)	0.28 (0.19, 0.42)	6.7 (4.0, 11.5)
Feng ST	84	76 (63, 86)	89 (81, 94)	6.75 (3.80, 11.99)	0.27 (0.17, 0.43)	24.8 (10.5, 58.3)
Jiang YQ	85	77 (62, 89)	95 (82, 99)	14.30 (3.68, 55.55)	0.24 (0.14, 0.42)	59.5 (12.1, 291.7)
Li Y	84	68 (45, 86)	96 (82, 100)	19.09 (2.73, 133.61)	0.33 (0.18, 0.61)	57.9 (6.5, 516.1)
Ma X	77	87 (76, 95)	72 (62, 80)	3.07 (2.22, 4.24)	0.18 (0.09, 0.36)	17.3 (7.0, 42.6)
Ni M	84	83 (61, 95)	86 (70, 95)	5.78 (2.51, 13.30)	0.20 (0.08, 0.50)	28.5 (6.8, 119.7)
Peng J	77	78 (72, 84)	76 (66, 84)	3.22 (2.27, 4.56)	0.29 (0.22, 0.38)	11.1 (6.4, 19.5)
Song D	86	85 (80, 89)	87 (83, 90)	6.38 (4.90, 8.31)	0.17 (0.13, 0.24)	36.6 (22.9, 58.7)
Wang H	76	71 (54, 84)	79 (68, 87)	3.30 (2.10, 5.20)	0.37 (0.23, 0.61)	8.9 (3.8, 20.8)
Xu X	81	89 (82, 93)	77 (73, 82)	3.93 (3.21, 4.82)	0.15 (0.09, 0.23)	26.7 (15.2, 46.9)
Yang L	87	89 (77, 96)	86 (79, 91)	6.25 (4.19, 9.31)	0.13 (0.06, 0.28)	47.4 (18.1, 123.9)
Yao Z	95	90 (70, 99)	100 (85, 100)	40.77 (2.62, 634.99)	0.12 (0.04, 0.37)	351.0 (15.9, 1000.0)
Yu Y	95	95 (89, 99)	93 (84, 98)	14.32 (5.55, 36.94)	0.05 (0.02, 0.13)	294.0 (70.6, 1000.0)
Zhang R	74	82 (73, 89)	70 (63, 77)	2.75 (2.15, 3.51)	0.25 (0.16, 0.40)	10.8 (5.8, 20.3)
Zhang W	71	72 (58, 83)	70 (56, 82)	2.43 (1.56, 3.78)	0.40 (0.25, 0.63)	6.09 (2.7, 13.8)
Zhang X	73	70 (53, 84)	76 (60, 89)	2.97 (1.62, 5.45)	0.39 (0.23, 0.66)	7.6 (2.7, 21.3)
Zhang Y	80	83 (74, 89)	76 (66, 85)	3.52 (2.37, 5.21)	0.23 (0.15, 0.35)	15.6 (7.7, 31.5)
Zheng J	78	91 (79, 97)	67 (55, 78)	2.76 (1.94, 3.93)	0.14 (0.06, 0.33)	19.6 (6.9, 56.3)
Zhu YJ	81	81 (68, 91)	81 (71, 88)	4.25 (2.72, 6.64)	0.23 (0.13, 0.41)	18.2 (7.7, 43.4)

Data in parentheses are 95% CIs. *Published in 2019; ^†^Published in 2020.

ACC, accuracy; SEN, sensitivity; SPE, specificity; PLR, positive likelihood ratio; NLR, negative likelihood ratio; DOR, diagnostic odds ratio.

### Meta-regression

Considerable heterogeneity existed among the studies (overall *I²* 94%; 95% CI: 88, 99; *P* < 0.001). Similarly, the forest plots indicated high heterogeneity with *I^2^* values > 50% for sensitivity (I² 62%; 95% CI: 44, 80; *P*< 0.001) and specificity (*I²* 88%; 95% CI: 83, 92; *P* < 0.001). The proportion of heterogeneity likely due to the threshold effect was small (8%). Therefore, we recorded no evidence of a threshold effect. To identify the source of heterogeneity, we performed univariable meta-regression analysis. **Table 3** shows the results of univariable meta-regression and subgroup analyses to explore the influence of patient characteristics, imaging modality, and study characteristics on the pooled sensitivity and specificity estimates. The results showed that all investigative covariates contributed to the heterogeneity in the sensitivity analysis (*P* < 0.05). In addition, combined clinical factors, MRI, CT and number of participants contributed to the heterogeneity in the specificity analysis (*P* < 0.05).

**Table 3:** Univariable meta-regression and subgroup analyses.

Parameter	Category	No. of Studies	Sensitivity (%)	*P_1_*	Specificity (%)	*P_2_*
Design	retrospective	20	83 (80, 86)	0.04	81 (77, 86)	0.15
	prospective	2	88 (78, 99)		100 (100, 100)	
Combine clinical factors	Yes	12	84 (81, 88)	0.00	81 (74, 87)	0.00
	no	10	83 (78, 88)		86 (80, 93)	
MRI	Yes	9	84 (79, 88)	0.00	84 (77, 90)	0.00
	no	13	84 (80, 88)		83 (76, 89)	
CT	Yes	9	84 (79, 88)	0.00	80 (72, 88)	0.00
	no	13	84 (80, 88)		85 (79, 90)	
US	Yes	3	87 (80, 95)	0.02	87 (74, 100)	0.44
	no	19	83 (80, 86)		83 (78, 88)	
No. of participants	≥100	15	84 (81, 87)	0.00	80 (74, 85)	0.00
	<100	7	81 (74, 88)		91 (86, 97)	
QUADAS	QUADAS high risk	5	74 (66, 82)	0.00	87 (79, 95)	0.08
	QUADAS no high risk	17	85 (83, 88)		82 (77, 87)	

Data in parentheses are 95% CIs. MRI, magnetic resonance imaging; CT, computed tomography; US, ultrasound; QUADAS, quality assessment of diagnostic accuracy studies.

### Subgroup Analysis

In terms of research design, prospective studies (n=2) had higher sensitivity (88%; 95% CI: 78, 99) and specificity (100%; 95% CI: 100, 100) than retrospective studies (n=20; sensitivity, 83% [95% CI: 80, 86]; specificity, 81% [95% CI: 77, 86]). Regardless of whether radiomics was combined with clinical risk factors to construct a predictive diagnostic model, the sensitivity (84%; 95% CI: 81, 88 vs. 83%; 95% CI: 78, 88) was basically equivalent for both. However, radiomics alone had a slightly higher specificity (n=10; 86%; 95% CI: 80, 93) than radiomics combined with clinical risk factors (n=12; 81%; 95% CI: 74, 87).

In terms of different imaging modalities, US (n=3) had a higher sensitivity (87%; 95% CI: 80, 95) and specificity (87%; 95% CI: 74, 100) than CT (n=9; sensitivity, 84% [95% CI: 79, 88]; specificity, 80% [95% CI: 72, 88]) and MRI (n=9; sensitivity, 84% [95% CI: 79, 88]; specificity, 84% [95% CI: 77, 90]). On the other hand, the pooled sensitivity, specificity and AUC (0.89; 95% CI: 0.86, 0.91 vs. 0.88; 95% CI: 0.85, 0.91) estimates were similar among studies with CT or MRI.

In addition, the pooled sensitivity estimates were higher in studies with 100 participants or more (n=15; 84%; 95% CI: 81, 87) than in studies with fewer than 100 participants (n=7; 81%; 95% CI: 74, 88), but the pooled specificity (80%; 95% CI: 74, 85 vs. 91%; 95% CI: 86, 97) showed the opposite trend. Similarly, studies without high risk according to QUADAS (n=17) had a higher pooled sensitivity (85%; 95% CI: 83, 88) but a lower specificity (82%; 95% CI: 77, 87) than studies with high risk according to QUADAS (n=5; sensitivity, 74% [95% CI: 66, 82]; specificity, 87% [95% CI: 79, 95]).

## Discussion

Our meta-analysis showed high pooled sensitivity (84%; 95% CI: 81, 87), specificity (83%; 95% CI: 78, 87), and AUC (0.90; 95% CI: 0.87, 0.92) values. which demonstrated that radiomics has the potential ability to preoperatively differentiate MVI status in HCC. The confirmation of this evidence will be beneficial to the formulation of optimal preoperative therapeutic strategies for patients with MVI of HCC. For example, if the presence of MVI is confirmed preoperatively, the extent of resection or ablation will be expanded. So it has great clinical value in reducing recurrence and improving the survival rate of HCC patients.

Likelihood ratios and posttest probabilities can also provide important information about the likelihood that a patient with a positive or negative test actually has MVI or not. Through our meta-analysis, a PLR of 5 indicates that the test is five times more likely to correctly judge a positive result than incorrectly judge a positive result, and the posttest probability for a positive test result is 77%. Similarly, an NLR of 0.19 indicates that the test is 0.19 times more likely to incorrectly judge a negative result than correctly judge a negative result, and the posttest probability for a negative test result is 11%. These results further indicate that radiomics has important clinical value in preoperatively evaluating the MVI status in HCC.

A previous meta-analysis reported that the sensitivity, specificity, and AUC of MVI prediction in HCC were 78% (95% CI: 75, 80), 78% (95% CI: 76, 81) and 0.855 for radiomics and 73% (95% CI: 0.71, 0.75), 82% (95% CI: 80, 83) and 0.860 for non-radiomics, respectively (41). The results indicated that the diagnostic performance for predicting MVI status in HCC was equivalent between radiomics and non-radiomics. However, the results reported above were all lower than those of our meta-analysis. A reasonable interpretation is that the number of included studies was not enough for radiomics (n=9) in the previous meta-analysis. This meta-analysis included 22 studies, showing that radiomics had a higher performance than non-radiomics for the preoperative prediction of MVI status in HCC.

Substantial heterogeneity among the studies was observed, so we performed meta-regression and subgroup analyses to detect the sources of heterogeneity. Due to the limitation of the number of included studies, we only performed univariable meta-regression analysis instead of multivariable meta-regression analysis. The results showed that all the observed indicators contributed to the source of heterogeneity. In addition, each included study used a different methodological design, which was only a part of the heterogeneity, and it was not possible to find all sources of heterogeneity.

We used five key factors for subgroup analysis. In the study design subgroup analysis, prospective studies were better than retrospective studies. This result is reasonable given that prospective studies have a clear purpose, a thorough design, proper observational indicators and so on, suggesting that more prospective studies in the future will improve the predictive performance for MVI. However, only 2 prospective studies addressed the use of radiomics for the evaluation of MVI status in HCC, and more high-quality evidence is needed to reach more definitive conclusions. Some previous studies have shown that an MVI predictive model with combined clinical risk factors had higher diagnostic performance (26, 27, 42). However, the results of our meta-analysis showed that the combination of clinical risk factors with radiomics did not improve the diagnostic ability. This indicates that radiomics alone could also achieve high diagnostic performance, which was consistent with the results of other previous studies (14, 19, 33).

We performed subgroup analysis to compare the diagnostic performance of radiomics based on different imaging modalities. The results showed that CT and MRI were essentially equivalent, consistent with previous studies (41, 43). However, due to multiparameter imaging and hepatobiliary phase-specific imaging agents, MRI has greater advantages in the diagnostic sensitivity of HCC. For example, a prospective study by Granito A, et al. showed that the diagnostic sensitivity of the hepatobiliary phase for the diagnosis of small HCC was 100% (95% CI: 90-100) with Gd-EOB-DTPA enhanced magnetic resonance, which had a higher sensitivity than contrast-enhanced CT and US (44). In our meta-analysis, US was superior to CT or MRI, but only three studies focusing on grayscale US were included, and two of them were prospective studies. Additionally, another US study with a retrospective design reported an AUC of only 0.68 (17). So the pooled result is incompletely convincing showing ultrasound superior to CT or MRI. However, again, prospective studies can significantly improve the predictive performance. Only one study on PET-CT was not included in the subgroup analysis. In addition, the results showed that the studies with large samples and without a high risk of bias had higher sensitivity. Therefore, in future studies, increasing the sample size and reducing bias will improve the ability to identify MVI.

We recognize that our meta-analysis has several limitations. First, mostly retrospective studies were included in our analysis, and patient selection could introduce some bias. Second, all included studies were from China. Studies from other countries were excluded for various reasons; for example, 2 studies from the United States were excluded because we could not reconstruct the 2×2 contingency table (45, 46). Thus, some characteristic populations may have been missed, which could affect the general applicability of the results in clinical practice. Finally, although radiomics models aid in the identification of MVI, the modeling method used might affect the predictive results of radiomics analysis. Each included study would have resulted in a different radiomics model, so it does not currently elucidate a clear modeling method to determine presence of MVI. However, multiple modeling methods can be attempted to achieve optimal model selection.

## Conclusion

In summary, our meta-analysis demonstrated that radiomics is a promising noninvasive method, and it has high preoperative identification performance for MVI status, which has crucial guiding significance for surgical planning of HCC patients in clinical practice. CT and MRI had a comparable predictive performance for MVI, but US and PET-CT still need to be conducted in more studies for further analysis based on radiomics methods. Moreover, it is necessary to carry out additional prospective, large-scale and multicenter studies with radiomics methods to improve the preoperative diagnostic performance of MVI in the future.

## Data Availability Statement

The original contributions presented in the study are included in the article/**Supplementary Material**. Further inquiries can be directed to the corresponding author.

## Author Contributions

Study design: LL, CW, YH, JC, DY, and ZS. Literature search and study selection: LL, CW and YH. Data extraction and quality assessment: LL, CW, JC and ZS. Statistical analysis: LL, DY and YH. Study supervision: ZS and YH. ZS obtained the research fund. Editing and review of the manuscript: all authors. All authors contributed to the article and approved the submitted version.

## Funding

This study was supported by the National Natural Science Foundation of China (Grant No. 82072038) and the Natural Science Foundation of Guangdong Province, China (Grant No. 2018A0303130070).

## Conflict of Interest

The authors declare that the research was conducted in the absence of any commercial or financial relationships that could be construed as a potential conflict of interest.

## Publisher’s Note

All claims expressed in this article are solely those of the authors and do not necessarily represent those of their affiliated organizations, or those of the publisher, the editors and the reviewers. Any product that may be evaluated in this article, or claim that may be made by its manufacturer, is not guaranteed or endorsed by the publisher.
