# Supplementary material for: Radiomics for the Preoperative Evaluation of Microvascular Invasion in Hepatocellular Carcinoma: A Meta-Analysis
Source: Front Oncol. 2022 Apr 7;12:831996. doi: 10.3389/fonc.2022.831996 (PMC9021380; doi:10.3389/fonc.2022.831996)
Supplement: Supplementary file 1 [file Table_1.docx]

**Supplementary materials: Search strategy**

# Pubmed

#1. (("Liver Neoplasms"[Mesh]) OR "Carcinoma, Hepatocellular"[Mesh]) OR

(((((((((((((((((((((((Neoplasms, Hepatic) OR (Neoplasms, Liver)) OR (Liver

Neoplasm)) OR (Neoplasm, Liver)) OR (Hepatic Neoplasms)) OR (Hepatic

Neoplasm)) OR (Neoplasm, Hepatic)) OR (Cancer of Liver)) OR (Hepatocellular

Cancer)) OR (Cancers, Hepatocellular)) OR (Hepatocellular Cancers)) OR (Hepatic

Cancer)) OR (Cancer, Hepatic)) OR (Cancers, Hepatic)) OR (Hepatic Cancers)) OR

(Liver Cancer)) OR (Cancer, Liver)) OR (Cancers, Liver)) OR (Liver Cancers)) OR

(Cancer of the Liver)) OR (Cancer, Hepatocellular)) OR ((((((((((((((((((Carcinomas,

Hepatocellular) OR (Hepatocellular Carcinomas)) OR (Liver Cell Carcinoma, Adult))

OR (Liver Cancer, Adult)) OR (Adult Liver Cancer)) OR (Adult Liver Cancers)) OR

(Cancer, Adult Liver)) OR (Cancers, Adult Liver)) OR (Liver Cancers, Adult)) OR

(Liver Cell Carcinoma)) OR (Carcinoma, Liver Cell)) OR (Carcinomas, Liver Cell))

OR (Cell Carcinoma, Liver)) OR (Cell Carcinomas, Liver)) OR (Liver Cell

Carcinomas)) OR (Hepatocellular Carcinoma)) OR (Hepatoma)) OR (Hepatomas)))

OR (HCC))

#2. ((microvascular OR microvessel) AND (invasion OR emboli)) OR (MVI)

#3. (radiomic) OR (radiomics)

#4. #1 AND #2 AND #3

# **Embase**

#1. 'liver cancer'/exp

#2. 'neoplasms, hepatic'

#3. 'neoplasms, liver'

#4. 'liver neoplasm'

#5. 'neoplasm, liver'

#6. 'hepatic neoplasms'

#7. 'hepatic neoplasm'

#8. 'neoplasm, hepatic'

#9. 'cancer of liver'

#10. 'hepatocellular cancer'

#11. 'cancers, hepatocellular'

#12. 'hepatocellular cancers'

#13. 'hepatic cancer'

#14. 'cancer, hepatic'

#15. 'cancers, hepatic'

#16. 'hepatic cancers'

#17. 'liver cancer'

#18. 'cancer, liver'

#19. 'cancers, liver'

#20. 'liver cancers'#21. 'cancer of the liver'

#22. 'cancer, hepatocellular'

#23. 'carcinomas, hepatocellular'

#24. 'hepatocellular carcinomas'

#25. 'liver cell carcinoma, adult'

#26. 'liver cancer, adult'

#27. 'adult liver cancer'

#28. 'adult liver cancers'

#29. 'cancer, adult liver'

#30. 'cancers, adult liver'

#31. 'liver cancers, adult'

#32. 'liver cell carcinoma'

#33. 'carcinoma, liver cell'

#34. 'carcinoma, liver cell'

#35. 'cell carcinoma, liver'

#36. 'cell carcinomas, liver'

#37. 'liver cell carcinomas'

#38. 'hepatocellular carcinoma'

#39. hepatoma

#40. hepatomas

#41. hcc

#42. #1 OR #2 OR #3 OR #4 OR #5 OR #6 OR #7 OR #8 OR #9 OR #10 OR #11 OR

#12 OR #13 OR #14 OR #15 OR #16 OR #17 OR #18 OR #19 OR #20 OR #21

OR #22 OR #23 OR #24 OR #25 OR #26 OR #27 OR #28 OR #29 OR #30 OR

#31 OR #32 OR #33 OR #34 OR #35 OR #36 OR #37 OR #38 OR #39 OR #40

OR #41

#43. microvascular

#44. Microvessel

#45. #43 OR #44

#46. invasion

#47. emboli

#48. #46 OR #47

#49. #45 AND #48

#50. mvi

#51. #49 OR #50

#52. radiomic

#53. radiomics

#54. #52 OR #53

#55. #42 AND #51 AND #54

# **The Cochrane Library**

#1. MeSH descriptor: [Liver Neoplasms] explode all trees

#2. MeSH descriptor: [Carcinoma, Hepatocellular] explode all trees

#3. (liver)

#4. (hepato*)

#5. (hepatic)

#6. (cancer*)

#7. (tumor*)

#8. (neoplas*)

#9. (carcinoma*)

#10. (maligna*)

#11. #3 OR #4 OR #5

#12. #6 OR #7 OR #8 OR #9 OR #10

#13. #11 AND #12

#14. (HCC)

#15. #1 OR #2 OR #13 OR #14

#16. (microvascular)

#17. (microvessel)

#18. (invasion)

#19. (emboli)

#20. #16 OR #17

#21. #18 OR #19

#22. #20 AND #21

#23. (MVI)

#24. #22 OR #23

#25. (radiomic*)

#26. #15 AND #24 AND #25
